# A multicomponent intervention incorporating CYP2C19 genotype-guided therapy and integrated pharmaceutical care reduces hospitalizations and improves adherence in ischemic stroke

**DOI:** 10.3389/fneur.2026.1848480

**Published:** 2026-07-29

**Authors:** Dan Zhang, Zhongru Meng, Yufei Li, Yang Miao

**Affiliations:** Department of Pharmacy, The First People’s Hospital of Yancheng, Yancheng First Hospital Affiliated Hospital of Nanjing University Medical School, Yancheng, Jiangsu, China

**Keywords:** CYP2C19 gene, individualized treatment, integrated pharmaceutical care, ischemic stroke, medication adherence

## Abstract

**Background:**

In ischemic stroke patients, antiplatelet therapy faces challenges from CYP2C19 genetic variability and poor post-discharge medication adherence, yet the value of integrating genotyping with comprehensive pharmaceutical care remains unclear across different recurrence risk populations.

**Methods:**

This single-center, prospective, parallel-group, open-label, assessor-blinded randomized controlled trial enrolled 300 ischemic stroke patients to evaluate a complex pharmacist-led intervention incorporating CYP2C19 genotype-guided antiplatelet therapy (individualized group, *n* = 150) versus routine care (control group, *n* = 150).

**Results:**

In the individualized treatment group, the proportion of patients with intermediate or poor CYP2C19 metabolizer phenotypes who received antiplatelet therapy adjustments was significantly higher (48.72 and 77.27% vs. 10.00%, *p* < 0.001), and genotype was positively correlated with medication adjustment (*ρ* = 0.476, *p* < 0.001). After 1 year, negative binomial regression showed that the individualized group had significantly lower hospitalization frequency than the control group (IRR = 0.700, 95% CI: 0.491–0.999, *p* = 0.049), and ANCOVA revealed significantly higher medication adherence (adjusted mean difference = 0.533, 95% CI: 0.250–0.815, *p* < 0.001). Subgroup analysis stratified by ESRS score showed that the beneficial effect of individualized treatment on reducing hospitalization was more pronounced in high-risk patients (ESRS ≥ 3: IRR = 0.640, 95% CI: 0.425–0.965, *p* = 0.033), whereas the effect in the low-risk group (ESRS < 3) did not reach statistical significance. After multivariable adjustment, the between-group difference in adverse reactions was not statistically significant (OR = 0.543, 95% CI: 0.279–1.057, *p* = 0.072).

**Conclusion:**

A multicomponent intervention comprising CYP2C19 genotype-guided therapy and intensive pharmacist-led integrated pharmaceutical care significantly improved medication adherence and reduced hospitalization frequency, with more pronounced benefits observed in patients at high risk of stroke recurrence, without increasing the risk of adverse drug reactions. However, the specific contribution of genotyping alone cannot be isolated from the overall care package.

## 1 Introduction

Worldwide, stroke ranks as the second leading cause of death, contributing to 11.6% of all deaths in 2019 (1). The most common form of stroke, ischemic stroke, is marked by elevated incidence, recurrence, disability, and mortality (2), representing 62.4% of global stroke cases in 2019 (1). This type of stroke can lead to neurological deficits and long-term adult disability, imposing a substantial health and economic burden (3, 4). Standardized treatment strategies are crucial for reducing recurrence, disability, and mortality in stroke patients. Therefore, etiological assessment and screening are necessary for patients with ischemic stroke to implement corresponding treatment and prevention strategies based on different etiologies, with emphasis on individualization and precision in drug selection, particularly during anticoagulation, antiplatelet, and lipid-lowering therapy (5).

Genetic variation and gene polymorphisms are not only important pathogenic factors for chronic cardiovascular and cerebrovascular diseases but also major contributors to inter-individual variability in drug therapy for these conditions. These variations are primarily due to polymorphisms in genes encoding drug-metabolizing enzymes, receptors, transporters, and targets, potentially impacting pharmacokinetics and pharmacodynamics and heightening the risk of adverse effects. Some drugs are only suitable for patients with specific genotypes, while others require dose adjustments based on genotype. The effectiveness and safety of therapeutic drugs for ischemic stroke often differ considerably among individuals due to genetic factors and gene variations (6, 7).

Antiplatelet therapy is essential for preventing early recurrence and in-stent thrombosis after stent implantation. A regimen of dual antiplatelet therapy (DAPT), including aspirin and clopidogrel, is frequently endorsed as a cornerstone for secondary prevention of ischemic stroke or transient ischemic attack (TIA) (8–10). In the past, ischemic stroke patients’ antiplatelet therapy dosing largely followed average doses outlined by drug labels, clinical guidelines, or research literature. However, clinical practice has revealed significant variability in response to antiplatelet drugs such as clopidogrel and aspirin. Due to genetic differences and gene polymorphisms, significant individual variability exists in the clinical use of antiplatelet agents, with 5–40% of ischemic stroke patients showing no response to aspirin and 4–30% exhibiting poor response to clopidogrel (11).

Clopidogrel requires conversion to an active metabolite via hepatic cytochrome P450 (CYP) enzymes. The reversible oral P2Y12 receptor antagonist ticagrelor achieves antiplatelet effects without the need for metabolic activation and may offer similar or superior inhibition of platelet aggregation compared with clopidogrel (12, 13). Clopidogrel is less effective for secondary stroke prevention in patients with CYP2C19 loss-of-function alleles, present in 25% of Caucasians and up to 60% of Asians (6, 7, 14). Genotype data from the CHANCE trial indicated that clopidogrel-aspirin provided greater benefit for high-risk (ESRS≥3) carriers of CYP2C19 loss-of-function alleles (15). Evaluating stroke recurrence risk in patients with CYP2C19 loss-of-function alleles may be crucial for determining optimal antiplatelet therapy (15, 16).

Pharmacogenomics-based individualized pharmacotherapy can personalize the assessment of drug-related gene polymorphisms by analyzing patient genotypes for drug targets and metabolizing enzymes. This allows for individualized selection of drug types and doses based on relevant genotypes, thereby effectively improving drug efficacy and reducing adverse reactions. In Chinese patients with minor ischemic stroke or TIA who carry CYP2C19 loss-of-function alleles, treatment with ticagrelor in a multicenter randomized trial was associated with a slightly lower 90-day stroke risk compared with clopidogrel, while the incidence of moderate or severe bleeding remained similar (17).

Additionally, patients with ischemic stroke often have underlying diseases, facing challenges such as polypharmacy, poor adherence, suboptimal efficacy, and increased drug–drug interactions. Given the predominance of elderly patients, medication adherence tends to be even poorer. Clinical pharmacists are needed to provide individualized and standardized management of their pharmacotherapy to improve efficacy, reduce adverse reactions and costs, and prevent or minimize stroke recurrence (2). Integrated Pharmaceutical Care (IPC) is a novel pharmaceutical model and philosophy referring to the pharmaceutical services provided by pharmacists throughout the entire disease treatment process, including before, during, and after medication use. Specifically, pharmacists are responsible for drug use, guidance, and supervision, working toward the established goal of improving quality of life throughout the treatment process to ensure patients take medication safely and effectively and achieve optimal treatment outcomes (2).

However, current pharmaceutical services provided by clinical pharmacists for stroke patients, both domestically and internationally, are mostly confined to the hospital setting, with limited extension to post-discharge care. This often leads to a serious management gap after discharge, resulting in low medication adherence. In contrast, studies have shown that enhancing communication between hospital and community pharmacists, including the use of discharge medication summaries and bidirectional patient information sharing through an online tool (e.g., a secure messaging platform), effectively reduces medication discrepancies and helps improve medication continuity and safety in stroke survivors (18). Therefore, this study aims to establish and evaluate a novel integrated pharmaceutical care model based on CYP2C19 genotyping, investigating its impact on medication adherence, hospitalization risk, and safety in ischemic stroke patients, and analyzing its application value across populations with different stroke recurrence risks.

## 2 Materials and methods

### 2.1 Study design

This was a single-center, prospective, parallel-group, open-label, assessor-blinded randomized controlled trial evaluating a complex pharmacist-led intervention incorporating CYP2C19 genotype-guided antiplatelet therapy. The Ethics Committee of Yancheng First People’s Hospital approved the study protocol, and all patients provided informed consent prior to participation. This study was also filed with the National Medical Research Registration Information System^1^ under the filing number MR-32-26-036814.

### 2.2 Study subjects

This study included patients with ischemic stroke who were admitted to the Department of Neurology at Yancheng First People’s Hospital between January 2024 and December 2024. Inclusion criteria: age ≥ 18 years; ischemic stroke diagnosed per 2018 Chinese Guidelines and verified by CT or MRI; hospital stay ≥ 2 days; ability to read and understand the test questionnaire, or having an assistant to help. Exclusion criteria: age < 18 years; hospital stay < 2 days; cardioembolic or hemorrhagic cerebral infarction; severe liver or kidney dysfunction, malignant tumors, or other primary life-limiting diseases (e.g., advanced heart failure, severe chronic obstructive pulmonary disease); impaired consciousness, mental disorders, or communication difficulties.

### 2.3 Grouping and interventions

Using a random number table, 300 patients were allocated equally into a control group and an individualized treatment group, each comprising 150 participants.

Randomization and allocation concealment: The randomization sequence was generated by a clinical pharmacist who was not involved in patient eligibility assessment or outcome evaluation, using a computer-generated random number table (block randomization with a block size of 4) in SPSS. The allocation sequence was concealed in sequentially numbered, opaque, sealed envelopes. When a patient met the eligibility criteria and provided informed consent, another clinical pharmacist (who was responsible for patient enrollment and baseline data collection) opened the next envelope in sequence and assigned the patient to either the control group or the individualized treatment group.

Implementation: Enrollment and assignment were carried out by the clinical pharmacist responsible for patient enrollment and baseline data collection, who was not involved in patient eligibility assessment or outcome evaluation. The clinical pharmacists who delivered the individualized pharmaceutical care received the group assignment after the patient had been enrolled and the envelope was opened. Patients and the clinical pharmacists delivering the intervention could not be blinded to group assignment due to the nature of the intervention. However, outcome assessors were blinded to group allocation throughout the follow-up period.

Control Group: Received routine diagnostic and treatment services. During hospitalization, received routine medication guidance provided by ward nurses. After discharge, no proactive pharmaceutical follow-up was provided. Patients received scheduled telephone calls from trained interns, and the content of these calls was strictly limited to: inquiring about adverse drug reactions, asking about hospitalization events, general inquiries about the patient’s condition, and administering the study questionnaires. These calls did not include any medication counseling, adherence support, or personalized advice, nor were they made for the purpose of medication reconciliation or therapeutic monitoring. No other form of additional pharmaceutical service was provided by clinical pharmacists.

Individualized Treatment Group: Received integrated pharmaceutical care based on CYP2C19 genotyping in addition to routine diagnostic and treatment services. The service content covered the entire treatment process:

Pre-medication: Clinical pharmacists conducted a comprehensive assessment combining CYP2C19 genotyping results with patient clinical characteristics (e.g., age, liver and kidney function, comorbidities) to assist physicians in formulating individualized antiplatelet treatment plans (including selection and dose adjustment of clopidogrel or ticagrelor).

During Hospitalization: Clinical pharmacists provided comprehensive medication monitoring (defined as daily review of the patient’s medication list to identify drug–drug interactions, duplicate therapies, or inappropriate dosing; reconciliation of admission and discharge medications; and documentation of any medication-related problems), detailed medication education (including drug action, dosage, adverse reactions, and precautions), disease knowledge education (risk factors, lifestyle interventions), and psychological counseling. Standardized content of medication education covered: (1) drug name, indication, and expected benefits; (2) dosage and administration timing; (3) common adverse reactions (e.g., bleeding, dyspnea with ticagrelor) and when to seek medical attention; (4) importance of adherence and risks of discontinuation.

Post-discharge: Clinical pharmacists developed individualized medication education sheets in printed A4 format for patients, with instructions to attend pharmacist clinic follow-ups once a month. Pharmacists conducted monthly telephone follow-ups to assess patient risk factor control and medication adherence, performed medication reconciliation and education, and documented services provided. At the beginning of each telephone call, the pharmacist introduced themselves as “a clinical pharmacist from the hospital” and obtained verbal confirmation from the patient that they were willing to continue the conversation.

Study timeline: CYP2C19 genotyping was performed within 48 h of admission. After discharge, telephone follow-ups were conducted monthly. At each follow-up call, adverse drug reactions and hospitalization information were assessed. The medication adherence assessment was performed at month 12.

Details of the intervention protocol are provided in the Supplementary Study Protocol.

### 2.4 Pharmacist qualifications and training

Two clinical pharmacists (each with ≥3 years of experience in neurology) delivered the intervention. Both pharmacists completed a 4-h training session covering: (1) interpretation of CYP2C19 genotyping results; (2) the antiplatelet selection algorithm; (3) standardized medication education scripts; (4) telephone follow-up procedures; and (5) documentation requirements.

### 2.5 CYP2C19 genotyping

A 2 mL sample of peripheral venous blood was collected from patients using EDTA anticoagulation. The CYP2C19 genotype (including *2 and *3 alleles) was detected using the PCR-fluorescence probe method. Based on the results, patients were assigned to one of three metabolic phenotypes: normal metabolizers, carrying no loss-of-function alleles; intermediate metabolizers, carrying a single loss-of-function allele; and poor metabolizers, carrying two loss-of-function alleles. A standardized algorithm was used: for normal metabolizers, clopidogrel 75 mg daily was continued; for intermediate metabolizers, the dose of clopidogrel was increased or switched to ticagrelor; for poor metabolizers, unless contraindicated (e.g., history of intracranial hemorrhage, active bleeding, or severe dyspnea), clopidogrel was switched to ticagrelor 90 mg twice daily. All drug adjustments were made in consultation with the attending physician.

### 2.6 Outcome measures and evaluation criteria

Collected baseline information encompassed patient demographics (age, sex), medical history (hypertension, diabetes, myocardial infarction, peripheral arterial disease, atrial fibrillation, coronary heart disease), lifestyle factors (smoking, alcohol), NIHSS and ESRS scores, and laboratory values including homocysteine, LDL-C, total cholesterol, and triglycerides.

#### 2.6.1 Primary outcome measures

Classification of Hospitalization Frequency within One Year: Hospitalization events were identified from three sources: (1) our hospital information system; (2) patient self-report during follow-up calls; (3) when a patient reported hospitalization elsewhere, we requested discharge summaries from the respective hospital with patient consent. To minimize underascertainment, patients were specifically asked at each follow-up call, “Have you been admitted to any hospital (including other hospitals) since our last call?” Hospitalization was defined as rehospitalization due to stroke recurrence, confirmed by CT or MRI. Recurrence was defined as a new neurological deficit occurring after stabilization of the index stroke, consistent with the criteria used in the CHANCE trial. The frequency was classified as: 1 = 0–1 hospitalization, 2 = 2 hospitalizations, 3 = ≥3 hospitalizations.

Adherence to medication was measured with the 8-item Morisky Medication Adherence Scale (MMAS-8) (19). Adherence classification: <6 = poor, 6–7 = moderate, 8 = good.

#### 2.6.2 Secondary outcome measures

Antiplatelet Medication Adjustment: Documented whether antiplatelet treatment plans were adjusted during hospitalization or follow-up due to efficacy or safety issues.

Adverse Drug Reactions: Documented any adverse events potentially related to medication occurring during the follow-up period. Adverse events were collected through active questioning at each monthly follow-up call using a standardized checklist (“Since your last call, have you experienced any of the following: bleeding, bruising, shortness of breath, headache, stomach upset, or any other new symptoms?”). All reported events were verified against medical records when possible.

### 2.7 Statistical analysis

Statistical analysis was conducted with SPSS 31.0. Continuous variables were compared using independent *t*-tests, while categorical variables were presented as frequencies and percentages and analyzed with the χ^2^ test. For hospitalization frequency, negative binomial regression was used to compare groups. For the continuous MMAS-8 score, analysis of covariance (ANCOVA) was used as the primary analytical method, adjusting for potential confounders; a crude linear regression including only group assignment was performed as a sensitivity analysis. For adverse drug reactions, univariate logistic regression analyses were first performed for each candidate variable, and all variables were then entered simultaneously into a multivariable binary logistic regression model using the enter method. Missing data (MMAS-8: *n* = 4; hospitalization frequency: *n* = 2) were handled using complete case analysis, as the proportion of missingness was <3%. No patients were lost to follow-up. Differences with *p* < 0.05 were considered significant.

## 3 Results

### 3.1 Baseline characteristics comparison

This study included 300 patients, with 150 in the individualized treatment group and 150 in the control group. Comparisons between the two groups revealed no significant differences in age, sex, medical conditions (hypertension, diabetes, myocardial infarction, peripheral artery disease, atrial fibrillation, coronary heart disease), smoking and alcohol history, NIHSS and ESRS scores, or biochemical markers (HCY, LDL-C, total cholesterol, triglycerides) (*p* > 0.05), indicating that the groups were well-matched (Table 1).

**Table 1:** Baseline data of patients.

Variable	Control group (*n* = 150)	Individualized treatment group (*n* = 150)	*t/x* ^2^	*p*
Age	68.30 ± 0.93	66.37 ± 0.88	1.508	0.133
Male, n(%)	105 (70.00)	102 (68.00)	0.140	0.708
Hypertension, n(%)	118 (78.67)	113 (75.33)	0.471	0.493
Diabetes mellitus, n(%)	51 (34.00)	61 (40.67)	1.425	0.233
Prior myocardial infarction, n(%)	2 (1.33)	1 (0.67)	0.337	0.562
Peripheral arterial disease, n(%)	12 (8.00)	11 (7.33)	0.047	0.828
Atrial fibrillation, n(%)	5 (3.33)	7 (4.67)	0.347	0.556
Coronary heart disease, n(%)	11 (7.33)	10 (6.67)	0.51	0.821
Smoking history, n(%)	5 (3.33)	13 (8.67)	3.783	0.052
Alcohol consumption, n(%)	3 (2.00)	4 (3.33)	0.146	0.702
NIHSS score	4.33 ± 0.54	5.21 ± 0.68	−0.969	0.334
ESRS score	2.60 ± 1.35	2.47 ± 1.32	0.820	0.413
HCY (μmol/L)	15.44 ± 1.37	15.34 ± 0.68	0.069	0.945
LDL-C (mmol/L)	3.07 ± 0.53	2.70 ± 0.08	0.679	0.497
Total cholesterol (mmol/L)	4.31 ± 0.12	4.47 ± 0.10	−1.032	0.303
Triglycerides (mmol/L)	1.98 ± 0.21	1.82 ± 0.10	0.702	0.483

### 3.2 CYP2C19 genotype distribution and antiplatelet medication adjustment analysis

In the individualized treatment group, the distribution of CYP2C19 genotypes was as follows: normal metabolizers: 50 cases (33.33%), intermediate metabolizers: 78 cases (52.00%), poor metabolizers: 22 cases (14.67%). The distribution observed in this study is generally in agreement with previously reported carriage rates of CYP2C19 loss-of-function alleles in the Chinese population, approximately 60% (7, 14).

**Figure 1:**
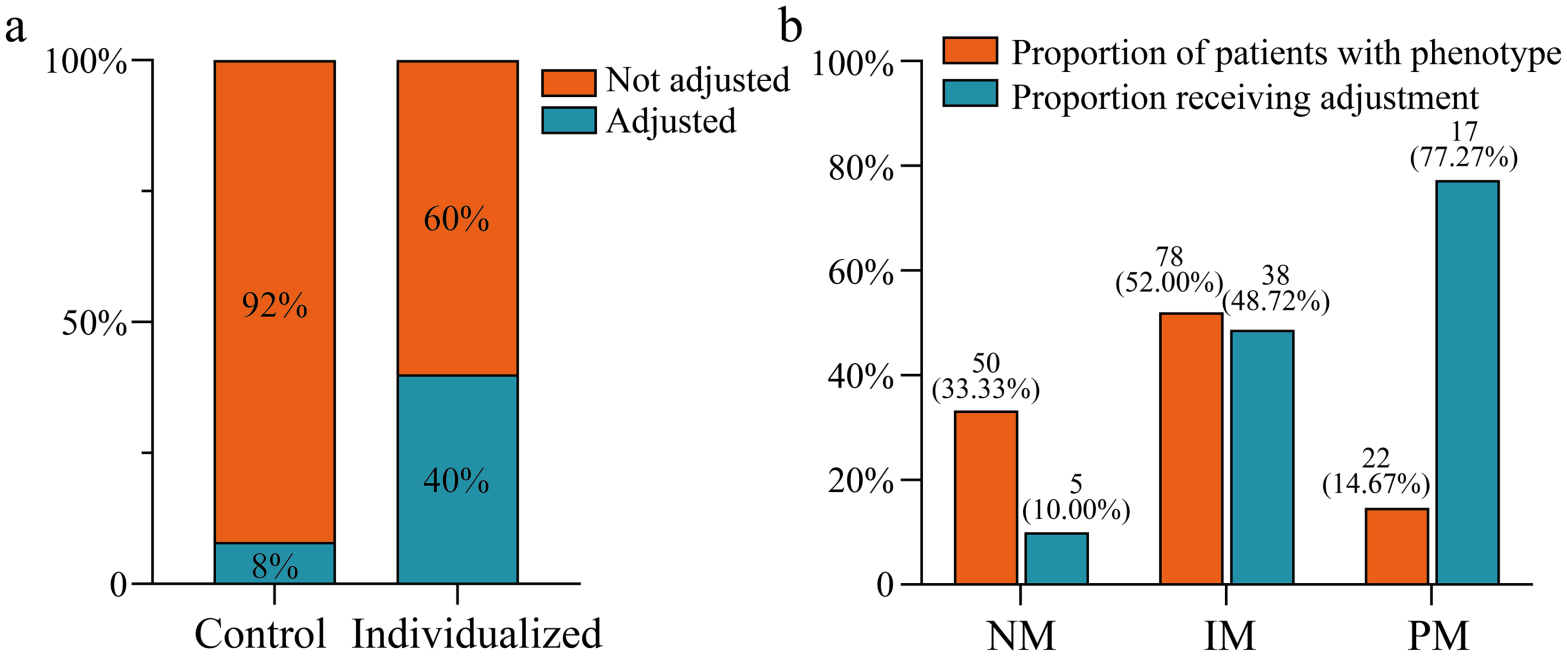
Antiplatelet medication adjustments across groups and CYP2C19 phenotypes. **(a)** Proportions of patients with antiplatelet medication adjustment in the control (*n* = 150) and individualized treatment (*n* = 150) groups. The adjustment rate was significantly higher in the individualized group (40%) than in the control group (8%). **(b)** CYP2C19 phenotype distribution and corresponding medication adjustment rates in the individualized group. NM: Normal metabolizer (*n* = 50); IM: Intermediate metabolizer (*n* = 78); PM: Poor metabolizer (*n* = 22). Percentages represent the proportion of patients who received antiplatelet medication adjustment within each CYP2C19 phenotype subgroup. Adjustment frequency increased stepwise from NM (10.00%) to IM (48.72%) to PM (77.27%) (*χ*^2^ = 33.955, *p* < 0.001). The stepwise increase was further supported by Spearman rank correlation analysis (*ρ* = 0.476, *p* < 0.001).

A significantly greater proportion of patients in the individualized treatment group (0.40 ± 0.492) underwent antiplatelet medication adjustments compared to the control group (0.08 ± 0.272), indicating enhanced genotype-guided therapy (*t* = −6.975, *p* < 0.001). As the metabolic phenotype transitioned from normal to poor, antiplatelet therapy adjustments were increasingly frequent across metabolic phenotypes, showing a significant stepwise trend: only 5 (10.00%) in the normal metabolizer group, 38 (48.72%) in the intermediate metabolizer group, and as high as 17 (77.27%) in the poor metabolizer group, with highly significant differences between groups (χ^2^ = 33.955, *p* < 0.001) (Figure 1). Spearman rank correlation analysis showed a significant positive association between CYP2C19 genotype and antiplatelet medication adjustments (*ρ* = 0.476, *p* < 0.001), suggesting that the likelihood of medication adjustment increased as the genotype shifted from normal to poor metabolizer. In most adjustment cases, patients with poor metabolizer phenotype switched from clopidogrel to ticagrelor, consistent with current guideline recommendations and the findings of the CHANCE-2 trial (9, 17).

### 3.3 Comparison of primary outcome measures

**Figure 2:**
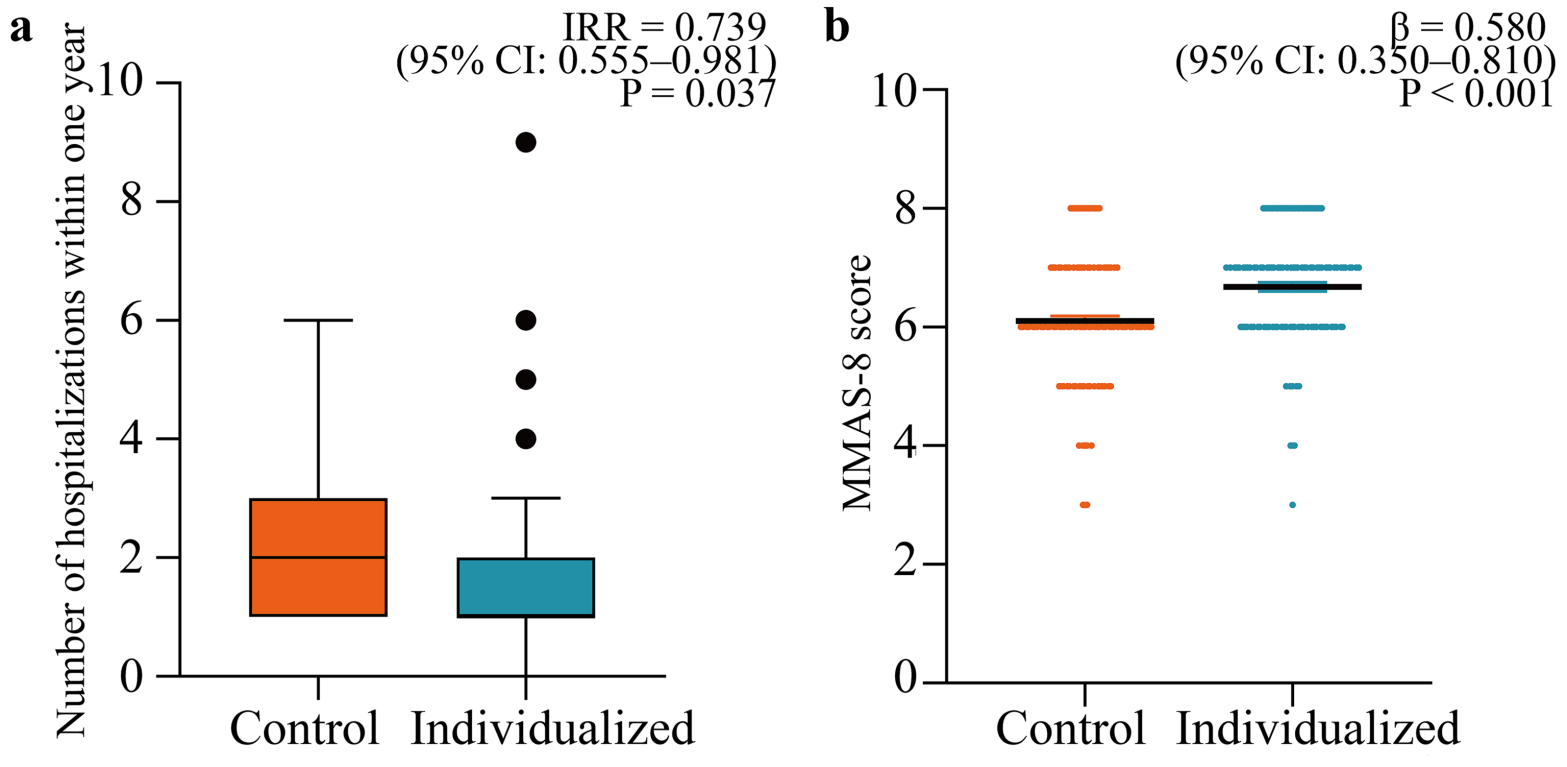
Clinical outcomes at 1-year follow-up. **(a)** Box plots of 1-year hospitalization counts in the control and individualized treatment groups. The individualized treatment group had significantly lower hospitalization frequency than the control group (negative binomial regression, unadjusted: *IRR* = 0.739, 95% *CI*: 0.555–0.981, *p* = 0.037). **(b)** Scatter plots showing individual MMAS-8 scores with group means (horizontal lines) in the control and individualized treatment groups. Unadjusted linear regression: *β* = 0.580 (95% *CI*: 0.350–0.810), *p* < 0.001.

After 1 year of follow-up, significant differences were observed in clinical outcome indicators between two groups. Negative binomial regression (unadjusted) showed that the individualized treatment group had significantly lower hospitalization frequency than the control group (IRR = 0.739, 95% CI: 0.555–0.981, *p* = 0.037) (Figure 2a). The mean hospitalization count was 1.49 ± 1.028 in the individualized group and 2.01 ± 1.023 in the control group. Crude linear regression including only group assignment showed that the individualized treatment group had significantly higher MMAS-8 scores than the control group (*β* = 0.580, 95% CI: 0.350–0.810, *p* < 0.001) (Figure 2b). The mean MMAS-8 score was 6.68 ± 0.944 in the individualized group and 6.10 ± 1.079 in the control group (based on 296 patients: 147 in the control group and 149 in the individualized group).

### 3.4 Multivariable negative binomial regression analysis of factors influencing hospitalization frequency

Using negative binomial regression with 1-year hospitalization count as the dependent variable, we constructed a multivariable model including study group, age, diabetes, atrial fibrillation, prior TIA/stroke history, and NIHSS score as covariates. As shown in Table 2, the individualized treatment group had significantly lower hospitalization frequency than the control group (IRR = 0.700, 95% CI: 0.491–0.999, *p* = 0.049). Age, diabetes, atrial fibrillation, prior TIA/stroke history, and NIHSS score were not significantly associated with hospitalization frequency.

**Table 2:** Multivariable negative binomial regression analysis of factors associated with 1-year hospitalization frequency.

Variable	*B*	SE	Wald *x*^2^	*P*	IRR (95%CI)
Study group	−0.357	0.181	3.866	**0.049**	0.700 (0.491–0.999)
NIHSS score	0.007	0.015	0.226	0.634	1.007 (0.978–1.037)
Age	0.013	0.009	2.193	0.139	1.013 (0.996–1.030)
Diabetes mellitus	0.216	0.184	1.369	0.242	1.241 (0.864–1.783)
Atrial fibrillation	−0.087	0.444	0.038	0.845	0.917 (0.384–2.188)
Prior TIA/ischemic stroke	0.293	0.210	1.947	0.163	1.340 (0.888–2.024)

*Bold indicates *p* < 0.05.

### 3.5 ANCOVA for MMAS-8 score

For medication adherence assessed by the MMAS-8 score, ANCOVA was performed adjusting for age, diabetes, NIHSS score, atrial fibrillation, and prior TIA/stroke history. As shown in Table 3, the individualized treatment group had significantly higher adherence scores than the control group (adjusted mean: 6.695 ± 0.094 vs. 6.163 ± 0.107; adjusted mean difference = 0.533, 95% CI: 0.250–0.815, *p* < 0.001). Among the covariates, diabetes was independently associated with lower adherence (*F* = 6.264, *p* = 0.013), suggesting that patients with diabetes may have lower adherence due to the need for more medications. Age (*p* = 0.295), NIHSS score (*p* = 0.619), atrial fibrillation (*p* = 0.254), and prior TIA/stroke history (*p* = 0.064) were not significantly associated.

**Table 3:** ANCOVA for MMAS-8 score.

Variable	Mean Square	*F*	*P*
Study group	13.515	13.803	**<0.001**
NIHSS score	0.242	0.247	0.619
Age	1.078	1.101	0.295
Diabetes mellitus	6.133	6.264	**0.013**
Atrial fibrillation	1.282	1.310	0.254
Prior TIA/ischemic stroke	3.398	3.470	0.064
Group	Adjusted Mean	SE	95%CI
Control	6.163	0.107	5.952–6.373
Individualized	6.695	0.094	6.510–6.881
Adjusted mean difference	0.533	0.143	0.250–0.815

Model adjusted for age, diabetes, NIHSS score, atrial fibrillation, and prior TIA/stroke. *R*^2^ = 0.128 (adjusted *R*^2^ = 0.100). *Bold indicates *p* < 0.05.

### 3.6 Subgroup analysis stratified by ESRS score using negative binomial regression

Patients were categorized into low-risk (ESRS < 3, *n* = 172) and high-risk (ESRS ≥ 3, *n* = 128) groups based on ESRS scores to examine the impact of multiple factors on hospitalization frequency across stroke recurrence risk populations. Subgroup analyses stratified by ESRS score were performed using negative binomial regression adjusting for study group, age, and prior TIA/stroke history. As shown in Table 4, in the high-risk group, the individualized treatment group had significantly lower hospitalization frequency than the control group (IRR = 0.640, 95% CI: 0.425–0.965, *p* = 0.033). In the low-risk group, the difference was not statistically significant (IRR = 0.777, 95% CI: 0.513–1.178, *p* = 0.236). Age and prior TIA/stroke history were not significantly associated with hospitalization frequency in either subgroup (all *p* > 0.05). These results indicate that the beneficial effect of the multicomponent intervention on reducing hospitalization was more pronounced in patients at high risk of stroke recurrence.

**Table 4:** Subgroup analysis of hospitalization frequency by ESRS score using negative binomial regression.

Variable	Low-risk group (ESRS<3)		High-risk group (ESRS≥3)	
	IRR (95%CI)	*P*	IRR (95%CI)	*P*
Study group	0.777 (0.513–1.178)	0.236	0.640 (0.425–0.965)	**0.033**
Age	1.004 (0.983–1.025)	0.732	1.004 (0.980–1.029)	0.741
Prior TIA/ischemic stroke	1.402 (0.748–2.632)	0.291	1.286 (0.860–1.922)	0.222

*Bold indicates *p* < 0.05.

### 3.7 Analysis of adverse drug reactions

Of the 300 patients, 63 (21.0%) reported adverse drug reactions during the 1-year follow-up. Univariate analyses showed that study group (OR = 0.498, 95% CI: 0.281–0.882, *p* = 0.017) and HAS-BLED score (OR = 1.394, 95% CI: 1.005–1.934, *p* = 0.047) were significantly associated with adverse reactions. Age, serum creatinine, diabetes, prior TIA/stroke history, and antiplatelet regimen category were not significantly associated in the univariate analyses (all *p* > 0.05) (Table 5). In the multivariable-adjusted model, after adjusting for age, serum creatinine, diabetes, HAS-BLED score, prior TIA/stroke history, and antiplatelet regimen category, the association between study group and adverse reactions was no longer statistically significant (OR = 0.543, 95% CI: 0.279–1.057, *p* = 0.072). HAS-BLED score remained independently associated with adverse reactions (OR = 1.785, 95% CI: 1.017–3.134, *p* = 0.044). These results indicate that the genotype-guided integrated pharmaceutical care model did not significantly increase the risk of adverse drug reactions, confirming its safety profile.

**Table 5:** Univariate and multivariate analysis of adverse drug reactions.

Variable	Univariate analysis		Multivariate analysis	
	OR(95%CI)	*P*	OR(95%CI)	*P*
Study group	0.498 (0.281–0.882)	**0.017**	0.543 (0.279–1.057)	0.072
Age	1.020 (0.993–1.047)	0.140	1.006 (0.971–1.042)	0.750
Serum creatinine	0.990 (0.977–1.003)	0.136	0.985 (0.970–1.001)	0.061
Diabetes	0.802 (0.446–1.443)	0.461	0.860 (0.436–1.697)	0.663
HAS-BLED score	1.394 (1.005–1.934)	**0.047**	1.785 (1.017–3.134)	**0.044**
Prior TIA/ischemic stroke	1.119 (0.609–2.056)	0.718	0.458 (0.175–1.197)	0.111
Antiplatelet regimen (ref. Clopidogrel)	0.697(0.453–1.073)	0.101	0.655 (0.419–1.024)	0.064

*Bold indicates *p* < 0.05.

## 4 Discussion

This study systematically established and evaluated a care model that combines CYP2C19 genotype-guided therapy with integrated pharmaceutical care for ischemic stroke patients. The results demonstrated that compared to routine treatment, this model significantly improved patient medication adherence and reduced hospitalization frequency within 1 year, with the effect being stronger among patients classified as high-risk for stroke recurrence (ESRS ≥ 3). Simultaneously, the model did not significantly elevate adverse drug reaction risk, indicating good safety. These findings provide strong evidence supporting individualized, integrated management of ischemic stroke. It should be noted, however, that the intervention package comprised both pharmacogenetic guidance and enhanced pharmacist-led services; therefore, the observed benefits cannot be attributed solely to CYP2C19 genotyping, but rather to the synergistic effect of the combined strategy.

This study first validated the central role of CYP2C19 genotype in clinical medication decision-making. In the individualized treatment group, up to 77.27% of poor metabolizers received antiplatelet therapy adjustments, significantly higher than normal metabolizers (10.00%) and intermediate metabolizers (48.72%). Spearman correlation analysis confirmed a dose–response relationship between genotype and medication adjustment. This result is highly consistent with multiple studies (6, 7, 14). Clopidogrel, as a prodrug, requires metabolism by hepatic CYP2C19 enzymes to its active form to exert its antiplatelet effect (7, 20). Patients with CYP2C19 loss-of-function alleles (such as *2 or *3) show markedly diminished enzyme activity, resulting in decreased active metabolite formation and suboptimal platelet inhibition, increasing the likelihood of stroke recurrence (6, 7, 21). In the genetic substudy of the CHANCE trial, minor stroke or TIA patients with LOF alleles showed decreased response to clopidogrel combined with aspirin (7). The following CHANCE-2 trial demonstrated that, in this population, ticagrelor plus aspirin outperformed clopidogrel plus aspirin in preventing stroke within 90 days (17, 22). The US FDA has also issued a black box warning recommending alternative treatment strategies for CYP2C19 poor metabolizers and identifying these patients through genotyping (14). Therefore, in this study, clinical pharmacists used genotyping results to switch poor metabolizers from clopidogrel to ticagrelor, which does not require metabolic activation, or adjusted other antiplatelet regimens. This represents a crucial first step in achieving precision treatment and optimizing clinical outcomes.

However, relying solely on genotyping for a single medication choice is insufficient. Patients with ischemic stroke are often elderly with multiple underlying conditions. Lacking professional guidance after discharge, they frequently experience a “management gap,” leading to a sharp decline in medication adherence, a significant risk factor for stroke recurrence (2, 23). For individuals with acute minor ischemic stroke or TIA, the likelihood of recurrent stroke within 3 months is estimated at 5–10% (23–25). Therefore, sustained medication management and follow-up are crucial. This study integrated genotyping with integrated pharmaceutical care, extending pharmaceutical monitoring from the hospital to the post-discharge period, covering the entire patient treatment journey. Patients in the individualized treatment group received detailed medication education during hospitalization, individualized medication education sheets after discharge, and monthly pharmacist clinic follow-ups and telephone follow-ups. This systematic, continuous intervention model resulted in significantly higher MMAS-8 scores in this group compared to control (6.68 vs. 6.10). ANCOVA confirmed that the individualized treatment group had significantly higher adherence scores than the control group after adjusting for covariates (adjusted mean difference = 0.533, 95% CI: 0.250–0.815, *p* < 0.001). This finding aligns with previous studies showing that pharmacist interventions improve adherence (8, 26). Improved adherence directly impacts therapeutic outcomes; this study observed that the individualized treatment group, with better adherence, had significantly lower hospitalization frequency within 1 year. Negative binomial regression confirmed that the individualized treatment group had significantly lower hospitalization frequency than the control group (IRR = 0.700, CI: 0.491–0.999, *p* = 0.049). This observation is in agreement with pooled data from the CHANCE and POINT trials, showing that consistent antiplatelet treatment significantly decreases the likelihood of new vascular events following stroke (23). We believe that the combination of precise drug selection based on genotyping and the sustained improvement in adherence facilitated by integrated pharmaceutical care constitutes the dual guarantee for improving clinical outcomes with this model.

Subgroup analysis revealed the differential effects of integrated pharmaceutical care across populations with varying stroke recurrence risks, one of the most significant findings. After stratifying patients based on ESRS score, negative binomial regression showed that individualized treatment was associated with lower hospitalization frequency in both low-risk and high-risk groups, although the difference did not reach statistical significance in the low-risk group (IRR = 0.777, *p* = 0.236). Notably, this protective effect was more pronounced in the high-risk group (ESRS ≥ 3), where patients receiving individualized treatment had significantly lower hospitalization frequency compared to the control group (IRR = 0.640, *p* = 0.033), corresponding to a 36.0% reduction in hospitalization frequency. The lack of statistical significance in the low-risk group may be attributable to the relatively small sample size and the lower baseline event rate in this population, rather than a true absence of treatment effect. This finding carries significant clinical implications. The ESRS, derived from extensive clinical trial datasets, predicts the likelihood of stroke recurrence in patients with ischemic stroke without atrial fibrillation, supporting clinicians in assessing the risk of recurrent stroke and cardiovascular complications (27, 28). High-risk patients often have more complex prothrombotic states, more extensive large artery atherosclerosis, or more severe systemic inflammatory responses (29). Their response to antiplatelet therapy may be poorer, and their need for precision treatment is greater. Previous genotype data from the CHANCE trial indicated that clopidogrel-aspirin provided greater benefit for high-risk (ESRS≥3) CYP2C19 LOF carriers (15, 30). Subgroup analyses of the CAPRIE trial also showed that compared to aspirin, clopidogrel provided significant benefit for high-risk patients (annual recurrent stroke risk >4% assessed by ESRS) (27). Our results extend these findings by confirming that in high-risk populations, combining precision medication with integrated management maximizes therapeutic benefits and significantly reduces hospitalization risk. This suggests that in clinical practice, high-risk patients should be prioritized for identification and allocated more intensive and precise pharmaceutical care resources.

Regarding adverse drug reactions, although univariate analysis showed a lower incidence in the individualized treatment group, the difference disappeared after multivariate adjustment. This result can be interpreted from several angles. First, precision medication guided by genotyping, such as switching from clopidogrel to ticagrelor, may avoid the potential risks associated with ineffective therapy (20, 31). Previous research indicates that individuals with CYP2C19 loss-of-function alleles undergoing clopidogrel treatment have an elevated risk of stroke and composite vascular events in comparison to non-carriers (7). Identifying these patients through genotyping and adjusting treatment can avoid ineffective therapy and its potential risks. The CHANCE-2 trial also demonstrated that ticagrelor combined with aspirin did not markedly increase the risk of severe bleeding in CYP2C19 LOF carriers (17). In the ticagrelor–aspirin group, the overall incidence of adverse events and discontinuations—especially due to minor bleeding, dyspnea, or arrhythmia—was increased, in line with previous ticagrelor trials (32–34), this was not observed in our study. Second, patients in the individualized treatment group received closer pharmaceutical monitoring, allowing pharmacists to detect and manage minor adverse reactions promptly, preventing progression to serious events. Under the integrated pharmaceutical care model, clinical pharmacists, through monthly follow-ups, can understand patients’ post-medication responses in a timely manner, enabling early intervention and medication adjustment for minor adverse reactions, thereby reducing the risk of serious adverse events. However, the disappearance of the adjusted difference also cautions against simply concluding that the model “significantly reduces” adverse reactions. A more rigorous conclusion is that while the model improves efficacy, it does not compromise safety; its safety profile is acceptable.

The innovations of this study are: (1) It deeply integrated CYP2C19 genotyping technology with the concept of integrated pharmaceutical care, constructing a new model for ischemic stroke patient management covering the entire “in-hospital to post-discharge” cycle; (2) It systematically evaluated the impact of this model on hard clinical endpoints such as medication adherence and hospitalization frequency; (3) Through subgroup analysis, it quantitatively revealed the differential benefits of this model across populations with varying stroke recurrence risks, providing a basis for optimizing the allocation of clinical resources. The significant association between HAS-BLED score and adverse drug reactions (OR = 1.785, 95% CI: 1.017–3.134, *p* = 0.044) is clinically plausible, as patients with higher bleeding risk scores are inherently more susceptible to bleeding-related adverse events. Notably, the fact that the intervention did not increase adverse reactions even after rigorous adjustment for this strong confounder strongly supports the safety of the genotype-guided pharmaceutical care model.

Because the individualized treatment group received both genotype-guided therapy and enhanced pharmaceutical care simultaneously, whereas the control group received neither, the study design compares a bundled intervention with routine care. Consequently, the specific effect of CYP2C19 genotyping cannot be isolated. The beneficial outcomes should be interpreted as the result of the combined package, not as an effect of pharmacogenetic testing alone. This distinction is essential for clinical implementation and future research design. This study is subject to limitations. First, the trial registration was performed retrospectively, although the study itself was designed and initiated prospectively. While we prespecified all outcome measures and statistical analyses prior to data analysis, we recognize that the lack of prospective registration remains a methodological limitation, as it is considered a key safeguard against selective reporting and analytical flexibility in randomized controlled trials. We acknowledge this limitation and emphasize the importance of prospective trial registration in future studies. Second, as a single-center study, the sample size is relatively limited, and the subjects originated from the same region, potentially limiting the generalizability of the results due to differences in population genetic backgrounds and healthcare resource accessibility. Third, the observation period was only 1 year; the impact on long-term clinical outcomes (e.g., cardiovascular and cerebrovascular mortality, long-term disability rates) requires confirmation through longer follow-up. Fourth, monitoring of adverse drug reactions relied on patient reports and medical records, which may have resulted in underreporting.

This study provides a new approach for the chronic disease management of ischemic stroke. Future research can be deepened in the following areas: First, To verify the generalizability and sustained benefits of this model, it is essential to conduct multicenter studies with larger sample sizes and long-term follow-up. Second, explore incorporating other pharmacogenomic markers (e.g., SLCO1B1 with statins, VKORC1 with warfarin) into this service model to achieve more comprehensive individualized treatment (24, 35). Third, integrate mobile health technologies (e.g., apps, smart medication dispensers) to enhance the accessibility and efficiency of pharmaceutical services, particularly for patients in remote areas. Fourth, perform health economic evaluations to quantify the value of this model in reducing rehospitalization rates and overall medical costs, providing evidence for policy-making.

## 5 Conclusion

This study demonstrates that a multicomponent intervention incorporating CYP2C19 genotype-guided antiplatelet therapy and intensive pharmacist-led pharmaceutical care significantly improves medication adherence and effectively reduces hospitalization frequency in patients with ischemic stroke, with more pronounced benefits in those at high risk of recurrence, while demonstrating good safety. While the intervention includes pharmacogenetic testing, the current design does not allow isolation of the specific effect of genotyping from the overall care package. Our findings support the implementation of such combined precision-management strategies in secondary prevention, but further studies are needed to clarify the independent contribution of each component.

## Data Availability

The authors acknowledge that the data presented in this study must be deposited and made publicly available in an acceptable repository, prior to publication. Frontiers cannot accept a manuscript that does not adhere to our open data policies.

## Footnotes

1
www.medicalresearch.org.cn
