# Supplementary material for: A multicomponent intervention incorporating CYP2C19 genotype-guided therapy and integrated pharmaceutical care reduces hospitalizations and improves adherence in ischemic stroke
Source: Front Neurol. 2026 Jul 29;17:1848480. doi: 10.3389/fneur.2026.1848480 (PMC13461525; doi:10.3389/fneur.2026.1848480)
Supplement: Supplementary file 1 [file Table_1.doc]

**Research Protocol: Construction and Application of a Genotyping-Based Integrated Pharmaceutical Care Model for Ischemic Stroke Patients**

Sponsor Unit: Yancheng First People's Hospital

Responsible Department: Department of Pharmacy

Project Leader: Yang Miao

Contact Number: +86 182 6190 7951

Version No.: V1.0

Version Date: January 1, 2024

**一、Rationale**

**1.1 Increasing incidence of cerebrovascular disease and the need for standardized treatment**

With population aging, deaths caused by chronic cardiovascular diseases such as coronary heart disease and cerebrovascular disease have been rising annually, becoming the leading cause of death in China. In 2020, cardiovascular diseases accounted for 48.00% and 45.86% of all deaths in rural and urban areas, respectively. According to surveys, there are 330 million patients with cardiovascular and cerebrovascular diseases in China, including 13 million with stroke and 11.39 million with coronary heart disease^[1]^. According to the China Health Statistics Yearbook 2022, cerebrovascular disease mortality accounted for 21.71% and 23.62% of total deaths in urban and rural areas, respectively, ranking among the top three causes of death. Ischemic stroke and transient ischemic attack (TIA) are the most common types of cerebrovascular disease, accounting for over 80%^[2]^. Data from GBD 2019^[3]^ show that in 2019, the total number of incident ischemic stroke cases in China was 2.87 million, an increase of 226.5% compared with 1990; the age-standardized incidence rate increased by 35%. The disease burden is becoming increasingly severe, and prevention/control efforts are critical.

Ischemic stroke is characterized by high incidence, high recurrence, high disability, and high mortality, with an annual recurrence rate of approximately 9.6%–17.7%^[4]^. Standardized treatment strategies are crucial for reducing recurrence, disability, and death. Because ischemic stroke has multiple causes, accurate etiological classification is essential for acute management, secondary prevention, and prognosis. Different etiologies require different treatment and prevention strategies. Moreover, drug selection must emphasize individualization and precision, especially during anticoagulation, antiplatelet therapy, and lipid-lowering treatment^[5]^. In addition, ischemic stroke patients often have underlying chronic diseases, requiring multiple medications, leading to poor adherence, suboptimal efficacy, and increased drug–drug interactions. Many are elderly with poor medication adherence, necessitating individualized and standardized pharmaceutical care by professional pharmacists to improve treatment outcomes, reduce adverse reactions and costs, and prevent or reduce stroke recurrence.

**1.2 Genotyping-guided precision pharmacotherapy for ischemic stroke becomes a new trend**

Extensive evidence from evidence-based medicine^[6]^ indicates that genetic variants and polymorphisms are not only important risk factors for chronic cardiovascular diseases but also major causes of interindividual variability in drug response. This variability arises mainly from polymorphisms in drug-metabolizing enzymes, receptors, transporters, and drug targets, which can affect pharmacokinetics and pharmacodynamics and increase the risk of adverse events. Some drugs are indicated only for patients with specific genotypes or require dose adjustment based on genotype. While standard doses work for most patients, a minority of “ultrarapid metabolizers” or “poor responders” may experience harm. Due to genetic factors and polymorphisms, the efficacy and safety of drugs used for ischemic stroke often vary among individuals.

Antiplatelet therapy plays an important role in preventing early recurrence and in-stent thrombosis after stenting. Traditionally, antiplatelet regimens for ischemic stroke are based on drug labels, guidelines, or average recommended doses. However, clinical practice has found considerable variability in response to clopidogrel, aspirin, and other antiplatelet agents. Due to genetic differences and polymorphisms, there is significant interindividual variability in antiplatelet drug use; 5%–40% of ischemic stroke patients are unresponsive to aspirin, and 4%–30% show poor response to clopidogrel^[7]^. Genotype data from the CHANCE trial indicated that clopidogrel–aspirin provided greater benefit in high-risk (ESRS ≥3) patients carrying CYP2C19 loss-of-function (LOF) alleles. This suggests that stratifying patients with CYP2C19 LOF alleles based on stroke recurrence risk may be important for selecting optimal antiplatelet therapy^[8]^.

Furthermore, statins are one of the cornerstones of secondary stroke prevention. Research^[9]^ shows that single nucleotide polymorphisms in the solute carrier anion transporter polypeptide-encoding gene SLCO1B1 are a genetic factor for statin-induced myopathy. The SLCO1B15 mutation reduces the transport activity of organic anion transporting polypeptide, impairing statin transport from plasma to the liver, resulting in elevated plasma statin concentrations and an increased risk of rhabdomyolysis or myopathy. In addition, apolipoprotein E carrying the APOE4 allele has increased affinity for LDL receptors, leading to feedback inhibition of LDL receptor expression, thereby reducing statin efficacy, often causing poor adherence and discontinuation. Therefore, selecting the appropriate statin and dose based on SLCO1B15 and APOE polymorphisms is feasible. Pharmacogenomics-based individualized pharmacotherapy can evaluate drug-related gene polymorphisms, enabling personalized selection of drug type and dose, thereby improving efficacy and reducing adverse reactions.

**1.3 Necessity of establishing an individualized, integrated pharmaceutical care model for ischemic stroke patients**

Currently, pharmaceutical care provided by clinical pharmacists to stroke patients mostly follows the American College of Clinical Pharmacy (ACCP) clinical pharmacist practice standard, i.e., the traditional pharmaceutical care model, which involves collaborating with other medical professionals to design, implement, and monitor patient medication regimens and participate in treatment management. This model is largely hospital-based and rarely extends to outpatient or post-discharge settings. Traditional pharmaceutical care often results in a serious management gap after discharge, low medication adherence, and, when recurrence or adverse reactions occur, physicians and pharmacists cannot promptly identify or intervene. Integrated pharmaceutical care (IPC) is a novel model and philosophy in which pharmacists provide pharmaceutical services before, during, and after drug administration throughout the entire disease treatment process. Specifically, it refers to pharmacists taking responsibility for drug use, guidance, and supervision around the goal of improving quality of life, throughout the disease treatment process, ensuring that patients take medications safely and effectively and achieve good therapeutic outcomes.

In recent years, a few reports have explored pharmaceutical care models for stroke patients. In China, Liao et al.^[10]^ examined integrated pharmaceutical care combined with psychological intervention for acute ischemic stroke patients, focusing on real-time prescription review, pharmaceutical monitoring, medication education, and prescription evaluation over only 4 weeks-insufficient to cover the entire treatment course. Ren et al.^[11]^ explored a precision medication pharmaceutical care pathway for ischemic stroke based on genotyping, incorporating improved pharmaceutical care systems, training, consultation clinics, or medication therapy management services, but did not develop a systematic, integrated care model. Therefore, finding a practical individualized, integrated pharmaceutical care model for ischemic stroke is key to reducing recurrence, especially in long-term post-discharge management.

**二、Research Objectives and Content**

**1. Objectives**

This project aims to establish and clinically apply a novel, risk-stratified, integrated pharmaceutical care model for ischemic stroke patients using precision techniques such as genotyping; to evaluate the application effects of integrated pharmaceutical care management in ischemic stroke patients with different Essen Stroke Risk Scores; and to provide individualized, standardized pharmaceutical care throughout the entire treatment process (in-hospital and post-discharge), improving medication safety, efficacy, and economy, reducing stroke recurrence risk, and alleviating social and family burdens.

**2. Research Content**

**1）Study Design**

Design type: Single-center, prospective, randomized, controlled trial.

Sample size: 300 patients in total, 150 in the control group, 150 in the individualized treatment group (using a random number table, block randomization with block size 4).

Study center: Department of Neurology, Yancheng First People's Hospital.

Study period: Enrollment from January 2024 to December 2024; follow-up for 12 months.

**2）Study Subjects**

Inclusion criteria:

Age ≥18 years;

Diagnosed with ischemic stroke according to the 2018 Chinese diagnostic criteria, confirmed by CT or MRI;

Hospital stay ≥2 days;

Able to read and understand the questionnaire, or with assistant support.

Exclusion criteria:

Age <18 years or hospital stay <2 days;

Cardiogenic embolism or hemorrhagic stroke;

Severe liver/kidney dysfunction, malignant tumor, or other life-limiting diseases (e.g., advanced heart failure, severe COPD);

Impaired consciousness, mental illness, or communication difficulties.

3) **Randomization and Blinding**

Random sequence generation: A clinical pharmacist not involved in patient eligibility assessment or outcome evaluation generated a computer random number table using SPSS (block randomization, block size 4).

Allocation concealment: Sequentially numbered, opaque, sealed envelopes.

Group assignment: After eligibility confirmation and signed informed consent, another clinical pharmacist responsible for enrollment opened the envelope and assigned the patient to the control or individualized treatment group.

Blinding: Patients and the intervention pharmacist cannot be blinded due to the nature of the intervention; outcome assessors (extracting inpatient data, administering MMAS-8 questionnaire) are blinded to group allocation.

4) **Interventions**

4.1) Control group

During hospitalization: Receive routine diagnostic and treatment services; routine medication instructions provided by ward nurses (only once at discharge, verbal information on drug name, dosage, and timing).

After discharge: No active pharmacy follow-up. Patients receive regular phone calls from trained interns, strictly limited to: inquiring about adverse reactions, hospitalization status, general condition, and completing study questionnaires. Calls do not include medication consultation, adherence support, individualized advice, medication reconciliation, or therapeutic monitoring.

4.2) Individualized treatment group

On the basis of routine diagnosis and treatment, patients receive integrated pharmaceutical services from clinical pharmacists based on CYP2C19 genotyping, covering the entire course:

Before medication: Pharmacists assist physicians in developing individualized antiplatelet regimens based on genotype results and clinical characteristics.

Standardized algorithm: For normal metabolizers, continue clopidogrel 75 mg daily; for intermediate metabolizers, increase clopidogrel dose or switch to ticagrelor; for poor metabolizers, unless contraindicated (e.g., history of intracranial hemorrhage, active bleeding, or severe dyspnea), switch from clopidogrel to ticagrelor 90 mg twice daily. All adjustments are made in consultation with the attending physician.

During hospitalization:

Comprehensive medication monitoring: daily verification of medication lists, identification of drug-drug interactions, duplicate medications, inappropriate doses; admission and discharge medication reconciliation; documentation of drug-related problems.

Standardized medication education: ①drug name, indication, expected benefits; ②dosage, administration timing; ③common adverse reactions (e.g., bleeding, ticagrelor-induced dyspnea) and when to seek medical attention; ④importance of adherence and risks of discontinuation.

Disease knowledge education (risk factors, lifestyle modifications) and psychological support.

After discharge:

Provide individualized medication education sheet (A4 paper version).

Monthly telephone follow-up: assess risk factor control, medication adherence, perform medication reconciliation and education. At the beginning of each call, the pharmacist introduces themselves and obtains verbal consent to continue.

**5) CYP2C19 Genotyping**

Blood collection: Within 48 hours of admission, collect 2 ml of peripheral venous blood (EDTA anticoagulant).

Detection method: PCR-fluorescence probe method to detect *2 and *3 alleles.

Metabolic phenotype classification:

Normal metabolizer: no loss-of-function (LOF) allele.

Intermediate metabolizer: one LOF allele.

Poor metabolizer: two LOF alleles.

**6) Study Timeline**

Genotyping completed within 48 hours of admission.

Monthly telephone follow-up after discharge.

Adverse reactions and hospitalization information assessed during each follow-up call.

Primary outcome assessment at month 12.

**7) Outcome Measures**

7.1) Primary outcomes

Annual hospitalization frequency grade: defined as rehospitalization due to recurrent stroke (confirmed by CT/MRI).

Data sources: hospital information system, patient self-report (asked at each follow-up: "Have you been hospitalized at any hospital?"), and with patient consent, discharge summaries obtained from other hospitals.

Grading: 1 = 0-1 time, 2 = 2 times, 3 = ≥3 times.

Medication adherence: assessed using the 8-item Morisky Medication Adherence Scale (MMAS-8).

Grading: <6 = poor, 6-7 = moderate, 8 = good.

7.2) Secondary outcomes

Proportion of antiplatelet drug adjustments.

Adverse drug reactions (active inquiry using standardized checklist: bleeding, bruising, shortness of breath, headache, stomach discomfort, etc., verified with medical records).

**8) Data Collection and Management**

Baseline data: age, sex, medical history (hypertension, diabetes, myocardial infarction, peripheral artery disease, atrial fibrillation, coronary heart disease), smoking/alcohol history, NIHSS score, ESRS score, homocysteine, LDL-C, total cholesterol, triglycerides.

Follow-up data: monthly telephone collection of hospitalization events, adverse reactions; MMAS-8 collected at month 12.

Missing data and loss to follow-up: Expected loss to follow-up rate is approximately 5%–10%. For patients who withdraw early or are lost to follow-up, we will collect outcome data at the time of withdrawal whenever possible, report reasons for loss, and compare baseline characteristics between the lost-to-follow-up and completed follow-up groups to assess attrition bias. For missing data, different strategies will be adopted depending on the missing proportion: if the missing proportion for primary outcomes is <5%, complete-case analysis will be used; if >5%, multiple imputation or last observation carried forward will be used for sensitivity analysis. Primary analysis will follow the intention-to-treat principle, including all randomized patients; missing outcome data will be handled as described. All missing data handling methods will be detailed in the final report.

**9) Statistical Analysis**

Software: SPSS 31.0.

Descriptive statistics: continuous variables as mean±standard deviation or median (IQR); categorical variables as frequency (percentage).

Comparisons: independent t-test (continuous variables), chi-square test (categorical variables).

Multivariable analysis: ordinal logistic regression (hospitalization grade, adherence grade), binary logistic regression (adverse reactions).

Subgroup analysis: stratified by ESRS score (<3 low risk, ≥3 high risk).

Significance level: P < 0.05 (two-sided).

**10) Ethics**

Ethics approval: Approved by the Ethics Committee of Yancheng First People's Hospital (Approval No. 2023-K-145).

Informed consent: All patients provide written informed consent before participation.

**11) Pharmacist Qualifications and Training**

Intervention pharmacists: 2 clinical pharmacists, each with ≥3 years of experience in neurology.

Training content (4 hours): ①interpretation of CYP2C19 genotyping results; ②antiplatelet selection algorithm; ③standardized medication education script; ④telephone follow-up procedures; ⑤documentation requirements.

**12) Funding Support**

Yancheng Health Commission Research Projects (YK2023057, YK2024034)

Jiangsu Pharmaceutical Association Project (202564033)

Yancheng Science and Technology Project (YCBK2024068)

**13) Supplementary Table**

**Attachment 1: Basic Information Collection Form for Ischemic Stroke Patients**

Medical ID：	Interviewer：	Date：	Y	M	D

| **Basic Information** | | | | | | | | | | | | | | | | | | |
| --- | --- | --- | --- | --- | --- | --- | --- | --- | --- | --- | --- | --- | --- | --- | --- | --- | --- | --- |
| Name | |  | | Gender | □M □F | | | Date of Birth | | | | | Y M D | | | Ethnicity | |  |
| Mailing Address | |  | | | | | | | | | | | Postal Code | | |  | | |
| Contact Phone | |  | | Mobile |  | | | Email | | | | |  | | | | | |
| Occupation | | □Worker □Farmer □Civil Servant □Freelance □Student □Military  □Businessperson □Staff □Other | | | | | | | | | | | | | | | | |
| Education Level | | □No schooling □Primary school □Junior high school □High school/Vocational school □Associate/Bachelor's degree □Master's degree or above | | | | | | | | | | | | | | | | |
| Average Monthly Income | | □<1000 RMB □1000–3000 RMB □3000–5000 RMB □>5000 RMB | | | | | | | | | | | | | | | | |
| Payment Method | | □Medical insurance □Commercial insurance □Self-pay □Public health insurance □Major illness fund □New Rural Cooperative Medical Scheme  □Other | | | | | | | | | | | | | | | | |
| Height | | （cm） | | Weight | | （kg） | | | | | Blood Pressure | | SBP＿＿＿＿（mmHg）/DBP＿＿＿＿（mmHg） | | | | | |
| NIHSS Score | | □ Not done | | | | | | | Glasgow Coma Scale | | | | | □ Not done | | | | |
| MMSE Score | | □ Not done | | | | | | | MoCA Score | | | | | □ Not done | | | | |
| Biochemical Tests | | Were the following measured within 3 days： □ Yes □ No □ Not | | | | | | | | | | | | | | | | |
|  |  | TG  HDL-C  GLU  AST  CERA  HCY  CRP  INR | | （mmol/L）  （mmol/L）  （mmol/L）  （U/L）  （μmol/L）  （μmol/L）  （mg/L） | | | □ Not done  □ Not done  □ Not done  □ Not done  □ Not done  □ Not done  □ Not done  □ Not done | | | | | TCHO  LDL-C  ALT  BUN  UA  HbA1c  HsCRP | | （mmol/L）  （mmol/L）  （U/L）  （mmol/L）  （mmol/L）  %  （mg/L） | | | □ Not done  □ Not done  □ Not done  □ Not done  □ Not done  □ Not done  □ Not done | |
| Auxiliary Examinations | | ECG | | □ Normal □ Abnormal □ Not done | | | | | | | | Echocardiography | | □ Normal □ Abnormal □ Not done | | | | |
|  |  | Head CT | | □ Normal □ Abnormal □ Not done | | | | | | | | Head MRI | | □ Normal □ Abnormal □ Not done | | | | |
|  |  | Head MRA | | □ Normal □ Abnormal □ Not done | | | | | | | | TCD | | □ Normal □ Abnormal □ Not done | | | | |
|  |  | Carotid Ultrasound | | □ Normal □ Abnormal □ Not done | | | | | | | | Angiography | | □ Normal □ Abnormal □ Not done | | | | |
|  |  | CTA | | □ Normal □ Abnormal □ Not done | | | | | | | |  | | □ Normal □ Abnormal □ Not done | | | | |
| Diagnosis | | □ New cerebral infarction □ Transient ischemic attack (TIA) □ High-risk individual for stroke □ Non-high-risk individual | | | | | | | | | | | | | | | | |
| **Medical History** | | | | | | | | | | | | | | | | | | |
| History of stroke | □ Yes | | | □ No □ Unknown | | | | | | | | | | | | | | |
|  | Type of stroke：□ Ischemic □ Hemorrhagic □ Unknown  Number of previous strokes：□ 1 □ 2 □ 3 □ Unknown | | | | | | | | | | | | | | | | | |
| History of TIA | □ Yes | | | □ No □ Unknown | | | | | | | | | | | | | | |
|  | Number of previous TIAs： □ 1 □ 2 □ 3 □ Unknown | | | | | | | | | | | | | | | | | |
| Atrial fibrillation | □ Yes | | | □ No □ Unknown | | | | | | | | | | | | | | |
|  | Type： □ Chronic paroxysmal □ Chronic persistent □ Chronic permanent  Current status： □ Currently treated □ Not treated | | | | | | | | | | | | | | | | | |
| Coronary heart disease | □ Yes | | | □ No □ Unknown | | | | | | | | | | | | | | |
|  | Year of diagnosis： | | | | | | | | | | | | | | | | | |
| Diabetes | □ Yes | | | □ No □ Unknown | | | | | | | | | | | | | | |
|  | Duration： □ ﹤ 1y □ ﹥5y □﹥10y □﹥20y □ ﹥30y | | | | | | | | | | | | | | | | | |
| Hypertension | □ Yes | | | □ No □ Unknown | | | | | | | | | | | | | | |
|  | Duration： □ ﹤1y □ ﹥5y □ ﹥10y □﹥20y □ ﹥30y | | | | | | | | | | | | | | | | | |
| Hypercholesterolemia | □ Yes | | | □ No □ Unknown | | | | | | | | | | | | | | |
|  | Duration： □ ﹤1y □ ﹥5y □﹥10y □﹥20y □ ﹥30y | | | | | | | | | | | | | | | | | |
| Peripheral vascular disease | □ Yes □ No □ Unknown | | | | | | | | | | | | | | | | | |
| Ischemic eye disease | □ Yes □ No □ Unknown | | | | | | | | | | | | | | | | | |
| Sudden deafness | □ Yes □ No □ Unknown | | | | | | | | | | | | | | | | | |
| Periodontitis/oral disease | □ Yes □ No □ Unknown （Note: missing teeth, frequent bleeding） | | | | | | | | | | | | | | | | | |
| Smoking | □ Yes □No □ Former （quit >1 year） | | | | | | | | | | | | | | | | | |
| Alcohol use | □ Yes □No □ Unknown | | | | | | | | | | | | | | | | | |
| Family history of cerebrovascular disease | □ Yes □No □ Unknown | | | | | | | | | | | | | | | | | |
| **Medication History** | | | | | | | | | | | | | | | | | | |
| Drug Name | | | Dosage/Route | | | | | | | Drug Name | | | | | Dosage/Route | | | |
|  | | |  | | | | | | |  | | | | |  | | | |
|  | | |  | | | | | | |  | | | | |  | | | |
|  | | |  | | | | | | |  | | | | |  | | | |
|  | | |  | | | | | | |  | | | | |  | | | |
|  | | |  | | | | | | |  | | | | |  | | | |
|  | | |  | | | | | | |  | | | | |  | | | |
|  | | |  | | | | | | |  | | | | |  | | | |
|  | | |  | | | | | | |  | | | | |  | | | |
|  | | |  | | | | | | |  | | | | |  | | | |
|  | | |  | | | | | | |  | | | | |  | | | |
| Adverse Drug Reactions |  | | | | | | | | | | | | | | | | | |
| Remarks |  | | | | | | | | | | | | | | | | | |

**Attachment 2: Essen Stroke Risk Score（ESRS）**

| **Risk Factor** | **Points** |
| --- | --- |
| 65–75 years | 1 |
| >75 years | 2 |
| Hypertension | 1 |
| Diabetes | 1 |
| Prior myocardial infarction | 1 |
| Other cardiovascular diseases (excluding MI and atrial fibrillation) | 1 |
| Peripheral artery disease | 1 |
| Smoking | 1 |
| Prior TIA or ischemic stroke | 1 |
| Total |  |

**Attachment 3: Patient Medication Education Record Form**

| Name |  | Gender | 🞎M 🞎F | Age |  | Department |  |
| --- | --- | --- | --- | --- | --- | --- | --- |
| Patient ID |  | Contact Information |  | | | Diagnosis |  |
| History of drug allergy： 🞎None 🞎Yes (describe drug name, manifestations, management, and outcome) | | | | | | | |
| Main Medications and Dosage Regimens | | | | | | | |
| Drug Name | | | Dosage/Route | | Start Date | | Remarks |
|  | | |  | |  | |  |
|  | | |  | |  | |  |
|  | | |  | |  | |  |
|  | | |  | |  | |  |
| Main Content of Medication Education | | | | | | | |
| Patient’s Medication Questions and Answers  Q：  A： | | | | | | | |
| Pharmacist Signature： | | | Patient/Family Signature： | | | Date： | |

**Attachment 4: 8-Item Morisky Medication Adherence Scale (MMAS-8)**

| **Item** | **Score** | |
| --- | --- | --- |
| 1．Do you sometimes forget to take your medication? | 口0 point Yes | 口1 point No |
| 2．Over the past 2 weeks, have there been days when you forgot to take your medication? | 口0 point Yes | 口1 point No |
| 3．When you feel worse or experience other symptoms during treatment, do you reduce the dose on your own without telling the doctor? | 口0 point Yes | 口1 point No |
| 4．When you travel or leave home for a long time, do you sometimes forget to bring your medication? | 口0 point Yes | 口1 point No |
| 5．Did you take your medication yesterday? | 口0 point Yes | 口1 point No |
| 6．When you feel your symptoms have improved or disappeared, have you stopped taking your medication? | 口0 point Yes | 口1 point No |
| 7．Do you find it difficult to stick to your treatment plan? | 口0 point Yes | 口1 point No |
| 8．How often do you find it hard to remember to take your medication on time and at the correct dose? | 口1 point Never  口0.75 point Rarely  口0.5 point Sometimes  口0.25 point Often  口0 point All the time | |

Scoring instructions: Total score is the sum of all 8 items, range 0-8. Higher score indicates better adherence.Score 8 = good adherence, Score 6-7 = moderate adherence, Score <6 = poor adherence.

**Attachment 5: CYP2C19 Genotyping Results and Antiplatelet Adjustment Record Form**

| Patient ID | CYP2C19*2 | CYP2C19*3 | Metabolic Phenotype | Baseline Antiplatelet Regimen | Adjusted (Y/N) | Post-Adjustment Regimen | Adjustment Date | Reason for Adjustment | Recorder |
| --- | --- | --- | --- | --- | --- | --- | --- | --- | --- |
|  |  |  |  |  |  |  |  |  |  |
|  |  |  |  |  |  |  |  |  |  |
|  |  |  |  |  |  |  |  |  |  |
|  |  |  |  |  |  |  |  |  |  |
|  |  |  |  |  |  |  |  |  |  |
|  |  |  |  |  |  |  |  |  |  |
|  |  |  |  |  |  |  |  |  |  |

**Attachment 6: Adverse Drug Reaction (ADR) Record Form**

| Patient ID | Follow-up Month | ADR | Onset Date | Severity | Management Action | Drug Stopped/Changed (Y/N) | Relationship to Drug | Recorder |
| --- | --- | --- | --- | --- | --- | --- | --- | --- |
|  |  |  |  |  |  |  |  |  |
|  |  |  |  |  |  |  |  |  |
|  |  |  |  |  |  |  |  |  |
|  |  |  |  |  |  |  |  |  |
|  |  |  |  |  |  |  |  |  |
|  |  |  |  |  |  |  |  |  |
|  |  |  |  |  |  |  |  |  |

**Attachment 7: Hospitalization Event Record Form**

| Patient ID | Group | Admission Date | Hospital Name | Primary Reason for Admission | Stroke Recurrence (Y/N) | Was Recurrence Confirmed by CT/MRI? | Discharge Diagnosis | Data Source | Recorder |
| --- | --- | --- | --- | --- | --- | --- | --- | --- | --- |
|  |  |  |  |  |  |  |  |  |  |
|  |  |  |  |  |  |  |  |  |  |
|  |  |  |  |  |  |  |  |  |  |
|  |  |  |  |  |  |  |  |  |  |
|  |  |  |  |  |  |  |  |  |  |
|  |  |  |  |  |  |  |  |  |  |
|  |  |  |  |  |  |  |  |  |  |

**三、Study Timeline**

| Phase | Time Period | Main Tasks |
| --- | --- | --- |
| Preparation | Oct 2023-Dec 2023 | Study design, preparation of ethics materials, submission for ethical approval; training of research pharmacists and interns; design of case report forms, randomization envelopes, and telephone follow-up scripts |
| Patient Enrollment | Jan 2024-Dec 2024 | Screening of hospitalized ischemic stroke patients, signing of informed consent forms, randomization (150 in control group, 150 in individualized treatment group) |
| In-Hospital Intervention | During hospitalization after enrollment | Control group: routine care+medication instructions by ward nurses; Individualized treatment group: genotyping (within 48 hours of admission) + comprehensive medication monitoring+standardized medication education |
| Post-Discharge Follow-up | Jan 2024-Dec 2025 | Monthly telephone follow-up (interns responsible for calls to control group: content limited to reminders and questionnaires; clinical pharmacists responsible for calls to individualized treatment group: includes medication reconciliation, education, etc.) |
| Outcome Assessment | 12 months after discharge (final call) | Completion of MMAS-8 scale assessment; extraction of annual hospitalization frequency data from hospital information system and patient reports |
| Data Cleaning and Locking | Jan 2026 | Verification of all case report forms, handling of missing data (4 missing MMAS-8, 2 missing hospitalization grade), database locking |

1. **References**

[1]Summary of China Cardiovascular Health and Disease Report 2022[J]. Chinese Circulation Journal, 2023, 38(6): 583-612.

[2]Chinese Society of Neurology, Chinese Society of Neurology Cerebrovascular Disease Group. Chinese Guidelines for Secondary Prevention of Ischemic Stroke and Transient Ischemic Attack 2022[J]. Chinese Journal of Neurology, 2022, 55(10): 1071-1109.

[3]Ma Q, Li R, Wang L, et al. Temporal trend and attributable risk factors of stroke burden in China, 1990-2019: an analysis for the Global Burden of Disease Study 2019. Lancet Public Health. 2021 ,6(12):e897-e906.

[4]Pan Y, Li Z, Li J, . Residual Risk and Its Risk Factors for Ischemic Stroke with Adherence to Guideline-Based Secondary Stroke Prevention. J Stroke. 2021 Jan;23(1):51-60..

[5]Ay H, Benner T, Arsava EM, et al. A computerized algorithm for etiologic classification of ischemic stroke: the Causative Classification of Stroke System. Stroke.2007,38(11):2979-84.

[6]Wang S, Yuan J, Huang Q. Gene therapy and prevention of cerebrovascular diseases[J]. Electronic Journal of Translational Medicine, 2018, 5(3): 42-44.

[7]Kamarova M, Baig S, Patel H, et al. Antiplatelet Use in Ischemic Stroke. Ann Pharmacother. 2022 Oct;56(10):1159-1173.

[8]Wang A, Meng X, Tian X, et al. Ticagrelor Aspirin vs Clopidogrel Aspirin in CYP2C19 Loss-of-Function Carriers With Minor Stroke or TIA Stratified by Risk Profile. Neurology. 2023 Jan 31;100(5):e497-e504.

[9]	Cooper-DeHoff RM, Niemi M, Ramsey LB, et al. The Clinical Pharmacogenetics Implementation Consortium Guideline for SLCO1B1, ABCG2, and CYP2C9 genotypes and Statin-Associated Musculoskeletal Symptoms. Clin Pharmacol Ther. 2022 May;111(5):1007-1021. doi: 10.1002/cpt.2557. Epub 2022 Mar 11. PMID: 35152405; PMCID: PMC9035072.

[10]Liao XM, Huang WH. Effect of integrated pharmaceutical care combined with psychological intervention on treatment outcomes and negative emotions in patients with acute ischemic stroke[J]. Chinese Journal of Prescription Drugs, 2021, 19(7): 59-60.

[11]Ren YL, Yin XQ. Implementation pathway of precision medication pharmaceutical care for ischemic stroke patients based on genetic testing technology[J]. Modern Chinese Journal of Medicine, 2023, 25(3): 83-85.
